# Minor Tobacco Alkaloids as Biomarkers to Distinguish Combusted Tobacco Use From Electronic Nicotine Delivery Systems Use. Two New Analytical Methods

**DOI:** 10.3389/fchem.2022.749089

**Published:** 2022-06-01

**Authors:** Peyton Jacob, Lawrence Chan, Polly Cheung, Kristina Bello, Lisa Yu, Gideon StHelen, Neal L. Benowitz

**Affiliations:** Clinical Pharmacology Program, Division of Cardiology, Department of Medicine, University of California, San Francisco, San Francisco, CA, United States

**Keywords:** tobacco, e-cigarettes, biomarkers of exposure, tobacco alkaloids, liquid chromatography -tandem mass spectrometry

## Abstract

Biomarkers for the use of electronic nicotine delivery systems (ENDS) are desirable for studies of the health effects of electronic cigarettes and related devices. However, the aerosols inhaled from these devices do not contain substances that are unique to this class of products, *i.e*., substances that are not present in cigarette smoke or those that do not have common environmental or dietary sources. Consequently, identifying selective biomarkers for ENDS use remains a challenge. If co-use of conventional tobacco products can be definitively ruled out, then nicotine and its metabolites are suitable for assessing exposure. Self-reports from questionnaires are often used to obtain information on product use. But self-reports may not always be accurate, and are not amenable to obtaining quantitative information on exposure. An alternative approach is to use selective biomarkers for conventional tobacco products to definitively rule out their use. In this article, we describe two new LC-MS/MS methods for the minor tobacco alkaloids anabasine, anatabine, nicotelline, anatalline, and 4-(methylnitrosamino)-1-(3-pyridyl)-1-butanol (NNAL), a tobacco-specific nitrosamine metabolite, all biomarkers that are selective for the use of conventional tobacco products. Applications of these biomarkers in studies of ENDS use and dual use of ENDS and conventional tobacco products are also discussed.

## Introduction

During the past several years, a variety of new tobacco products and nicotine delivery devices have been introduced. These include ENDS, heated tobacco products such as Philip Morris’ IQOS and British American Tobacco’s Glo, and oral nicotine delivery products such as Zyn, On! and Velo. Of these, ENDS, in particular electronic cigarettes are the most widely used. Most but not all public health researchers are of the opinion that these new products are generally less harmful than conventional tobacco products. The extent to which they could reduce harm is unknown, largely because some adverse health effects, in particular cancer and chronic lung disease, take many years to develop. Therefore, thorough epidemiological studies have not been possible. Furthermore, dual use of ENDS with combusted cigarettes is commonplace, and the extent of reduced exposure and potential harm in ENDS users who continue to smoke cigarettes is difficult to assess. (Goniewicz et al., 2018; Borland et al., 2019; Piper et al., 2019; Smith et al., 2021) In short term studies of acute effects of novel products such as ENDS, (Hajek et al., 2017; St Helen et al., 2020a) it is important to determine recent use of tobacco products as well as the extent of dual use of both products. For exposure assessment, self-reported use of particular products can be useful, but they are of limited utility for obtaining quantitative data. In this regard, biomarkers of exposure are useful. Biomarkers would be especially important to assess recent dual use in clinical trials of novel products and for epidemiological studies of health effects. Specific biomarkers have not been identified for e-cigarettes and other ENDS, because the substances in these products are nicotine, solvents used to generate the aerosols (propylene glycol and glycerol), and flavoring compounds which are found in conventional tobacco products and have dietary sources as well. (Schick et al., 2017) Pyrolysis reactions transform components of the e-liquids into various products during aerosol formation, but as yet no pyrolysis products unique to ENDS have been identified that could serve as selective biomarkers. Consequently, other than self-reports, which are of limited value, the only viable approach is to use biomarkers specific to tobacco products to identify and estimate the extent of their use in people using ENDS.

**FIGURE 1:**
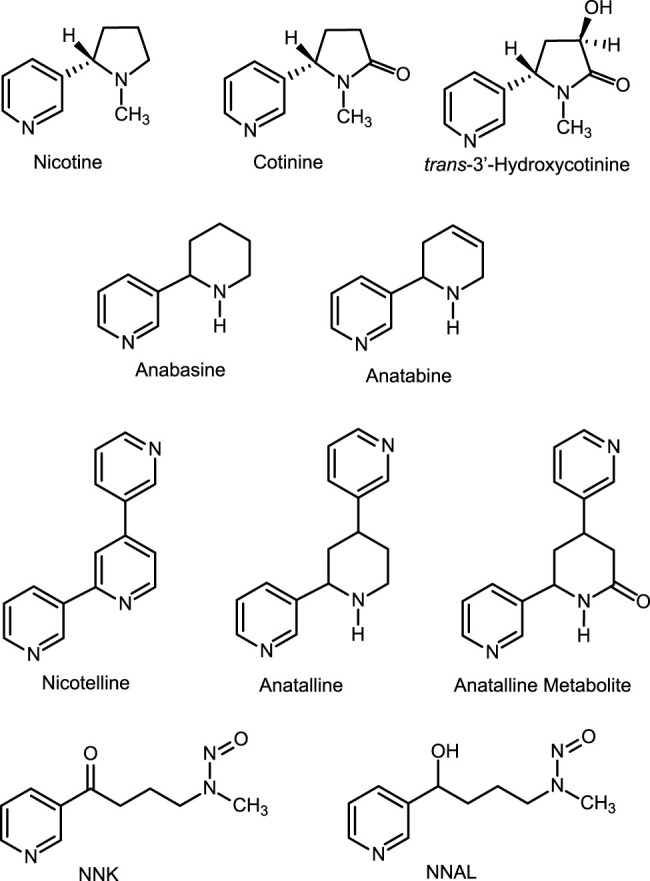
Biomarkers and biomarker metabolic precursors.

Tobacco contains a number of pyridine alkaloids other than the major alkaloid nicotine. (Schmeltz and Hoffmann, 1977; Rodgman and Perfetti, 2013) These minor alkaloids, which include anabasine, anatabine, anatalline, and nicotelline (Figure 1) are present in cigarette tobacco in concentrations ranging from about 1 to 1000 μg/g, compared to concentrations of about 15 mg/g for nicotine. (Jacob et al., 2013; Lisko et al., 2013) Although nicotine in nearly all e-liquids used in ENDS is derived from tobacco, the nicotine in most (but not all) products has been purified sufficiently that minor alkaloid concentrations are low compared to the amounts present in tobacco. (Palazzolo et al., 2019; Jacob et al., 2020) Consequently, minor alkaloids may be used as biomarkers for the use of conventional tobacco products in people using ENDS. (Berlin et al., 2019; Jacob et al., 2020) In this article we describe two new methods for quantitation of nicotine-related minor tobacco alkaloids in urine that can be used in this approach. One of these methods also measures concentrations of the tobacco-specific carcinogen metabolite 4-(methylnitrosamino)-1-(3-pyridyl)-1-butanol (NNAL). The goal of our studies was to develop methods to simultaneously quantify multiple biomarkers useful in studies of dual use of ENDS and combusted cigarettes.

**FIGURE 2:**
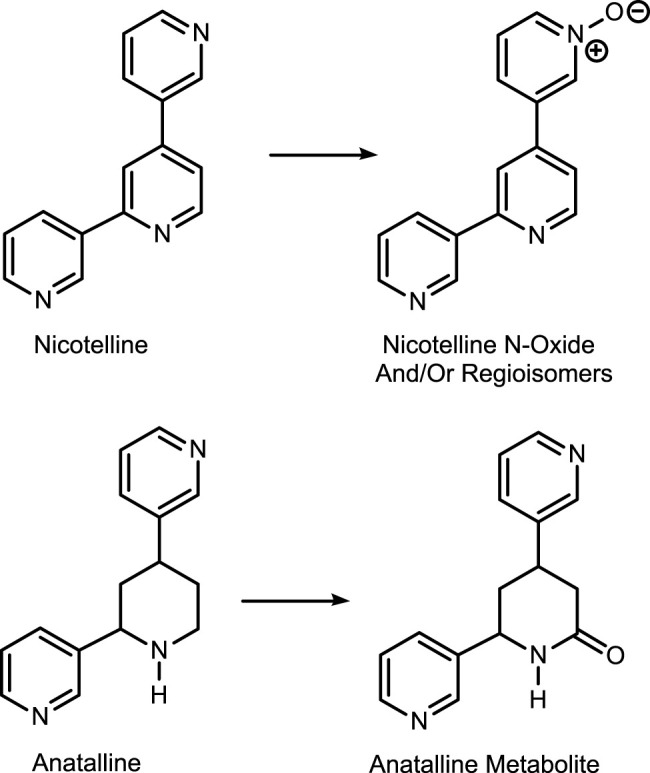
Metabolism of nicotelline and anatalline.

**FIGURE 3:**
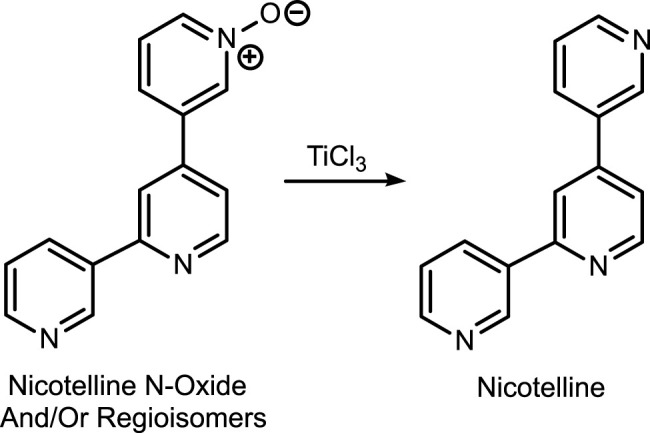
Reduction of nicotelline N-oxides prior to LC-MS/MS analysis.

**FIGURE 4:**
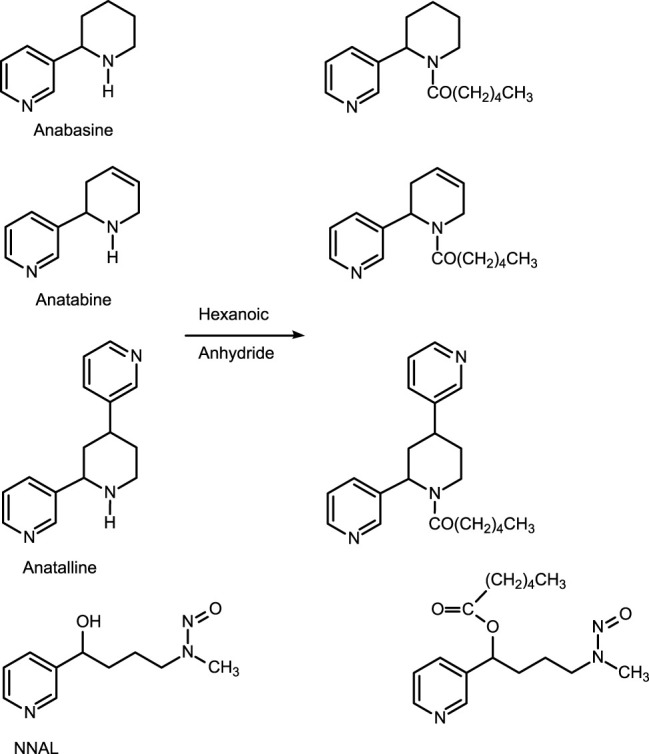
Derivatization of alkaloids and NNAL with hexanoic anhydride prior to LC-MS/MS analysis.

The first method is based on our published LC-MS/MS method for nicotelline, (Jacob et al., 2013) an alkaloid that we have proposed as a biomarker to distinguish ENDS use from use of combusted cigarettes. (Jacob et al., 2020) Since little if any nicotelline is excreted unchanged, and the metabolites identified so far are N-oxides (Figure 2), the method involves treating urine with titanium trichloride to convert the N-oxides back to nicotelline (Figure 3) that can be readily extracted and measured. Chromatography and mass spectrometry parameters were modified to include other minor tobacco alkaloid analytes. The second LC-MS/MS method utilizes a derivatization with hexanoic anhydride, developed for the carcinogen biomarker NNAL to enhance sensitivity. (Jacob et al., 2008) This derivatization also converts the secondary amine alkaloids anabasine, anatabine, and anatalline into amides, which results in improved chromatography, and allows simultaneous determination of these alkaloids with NNAL. (Figure 4) An advantage of these new methods is simultaneous determination of multiple biomarkers that have a wide range of biological half-lives, ranging from 2–3 h for nicotelline, to more than 10 days for NNAL. This can be important if measures of long-term exposure and recent exposure are desired. Another advantage is higher sensitivity (lower limits of quantitation) than previously reported methods, thus facilitating low-level exposure assessment. These advantages of the two new methods should make them especially useful in studies of dual use of ENDS and conventional tobacco products. We also introduce the alkaloid anatalline as a new, highly selective biomarker for tobacco exposure.

## Materials and Methods

### Reagents and Standards

Analytical standards and internal standards are available commercially (Toronto Research Chemicals, North York, ON, Canada, and other sources) or can be synthesized by published methods. (Surya Prakash Rao et al., 1997; Jacob et al., 2013) The analyte standards were anabasine (internal standard anabasine-d_4_, pyridine ring labelled), anatabine (internal standard anatabine-d_4_, pyridine ring labeled), nicotelline (internal standard nicotelline-d_8_, pyridine rings labeled), anatalline (internal standard anatalline-d_4_, pyridine ring labeled), anatalline metabolite: 4,6-di-3-pyridinyl-2-piperidinone (internal standard anatalline metabolite-d_4_, pyridine ring labeled), NNAL: 4-(methylnitrosamino)-1-(3-pyridinyl)-1-butanol (internal standard NNAL-d_3_, N-methyl labeled). Reagents and solvents used for sample extractions and for preparing LC mobile phases were of analytical reagent grade or HPLC grade.

### Instrumentation

LC-MS/MS analyses were carried out with a Thermo Accela UPLC pump and Pal Autosampler interfaced to a Thermo Vantage triple-stage quadrupole mass spectrometer, or with a Thermo/Dionex UltiMate 3000 RS Pump UPLC+ Focused UPLC and CTC/Dionex UltiMate 3000 XRS Open Autosampler interfaced to a Thermo Quantiva triple-stage quadruple mass spectrometer. Evaporations were carried out using a centrifugal vacuum evaporator, Thermo-Fisher Speedvac concentrator SPD 2010.

### Extraction Procedure, Method 1

The internal standards, in 0.01 N aqueous HCl, 100 μL of a mixture of anabasine-d_4_, (100 ng/ml) anatabine-d_4_ (100 ng/ml), nicotelline-d_8_ (10 ng/ml), anatalline-d_4_ (4 ng/ml) and anatalline metabolite-d_4_ (150 ng/ml), were added to 0.5 ml sample of urine. 100 μL titanium (III) chloride, 20% w/v solution in 2 N hydrochloric acid (ACROS Organics) were added to fortified urine sample, were mixed and incubated 30 min at room temperature. Saturated aqueous tetrasodium EDTA/concentrated ammonium hydroxide (4:1, 500 µL) was added next. Toluene/ethyl acetate (2:1, 4.5 ml) was added, the tubes were vortexed 5 min, centrifuged at 4,000 g for 10 min, and the aqueous phase frozen by immersion in a dry ice/acetone bath. The organic phase was poured to a new tube containing 0.5 ml 1 M sulfuric acid. The mixture was vortexed, centrifuged, and the upper layer was poured out and discarded after freezing the aqueous layer in dry ice/acetone. The acid phase containing the analytes was made basic with 0.5 ml 50% potassium carbonate, and 4 ml pentane/dichloromethane (1:1) was added. The mixture was vortexed, centrifuged, and placed in a dry ice/acetone bath to freeze the lower aqueous layer. The organic phase poured into a tube containing 100 µL10% HCl in methanol (to prevent evaporation of the analytes by converting them to non-volatile salts) before evaporating to dryness. The residues were reconstituted in 200 µL 200 mM ammonium formate in 10% MeOH that had been adjusted to pH 9 with concentrated aqueous ammonia. Standards and QC samples were prepared by spiking pooled non-smokers’ urine with the analytes, spanning the expected concentration ranges. QC sample concentrations were for anabasine, anatabine, and anatalline, in ng/mL: 30, 5, 0.25, 0.1, and 0 = blank urine matrix. For nicotelline they were, in ng/mL: 3, 0.5, 0.025, 0.01, 0 = blank urine matrix. For the anatalline metabolite they were, in ng/mL: 3, 0.5, 0 = blank urine matrix. Duplicate standards and QCs were extracted and analyzed with each sample run.

### Chromatography and Mass Spectrometry, Method 1

A 20 µL aliquot of the extract was injected *via* the autosampler into the LC-MS/MS system, Vantage or Quantiva system. Chromatography was performed on an X-Bridge BEH C18 column (2.5 µm particle size, 3 mm × 150 mm, Waters, United States) at 50°C with a flow rate of 0.6 ml/min, applying a gradient consisting of 20 mM ammonium formate in 10% methanol with pH 9 (A) and methanol (B). Preparation of 1 L of mobile phase A involves mixing 1.25 g of ammonium formate, 0.5 ml concentrated aqueous ammonia, 100 ml methanol and HPLC grade water to volume. Gradient conditions were as follows: 0 min: 100% A, 0–10 min: 100–40% A, 10–11 min: 40–0% A, 11–13 min: 0% A, 13–13.5 min: 0–100% A, 13.5–17 min: 100% A. Positive electrospray ionization (ESI) was used. The spray voltage was 3000, the vaporizer temperature was 450°C, the capillary temperature was 350°C, the sheath gas pressure was 45 psi, the auxiliary gas pressure was 5 psi, and the ion sweep gas pressure was 2 psi. The resolution of the first quadrupole, FWHM, was set at 0.4 amu, the resolution of the third quadrupole was set at 0.7 amu FWHM. The MS/MS system was run in the selected reaction monitoring (SRM) mode. Mass transitions for the analytes and internal standards are in Table 1.

**TABLE 1:** SRM transitions and collision energies (CE) for analytes and internal standards.

	Method 1			Method 2		
Analyte	Parent	Product	CE	Parent	Product	CE
Anabasine	163	146	14	261	120	30
Anabasine-d_4_	167	150	14	265	124	30
Anatabine	161	144	14	259	144	30
Anatabine-d_4_	165	148	14	263	148	30
Anatalline	240	197	18	338	197	30
Anatalline-d_4_	244	201	18	342	201	30
Anatalline Metabolite	254	195	25	NA	NA	NA
Anatalline Metabolite-d_4_	258	199	25	NA	NA	NA
Nicotelline	234	207	30	NA	NA	NA
Nicotelline-d_8_	242	214	30	NA	NA	NA
NNAL	NA	NA	NA	308	162	11
NNAL-d_3_	NA	NA	NA	311	165	11

### Data Analysis Method 1

The Thermo XCalibur/LC Quan software was used to generate calibration curves and calculate concentrations using peak area ratios of analyte/internal standard. Linear regression with 1/X weighting, “ignore origin” was used. Blanks (pooled non-smokers’ urine) were included in the standard curves and “ignore origin” was used to correct for the small amounts of analytes that might be present in non-smokers’ urine used to prepare standards, due to secondhand smoke exposure. Eight concentrations spanning the calibration range for each analyte were used, and standards were run in duplicate. Typically, one set of standards was injected at the beginning of the run, and one set following injection of the clinical study samples. Concentrations of the standards, equations and correlation coefficients for representative calibration curves are in the Supplementary Material document.

### Extraction Procedure, Method 2

The internal standards, in 0.01 N HCl, 100 μL of a mixture of anabasine-d_4_ (100 ng/ml), anatabine-d_4_ (100 ng/ml), anatalline-d_4_ (4 ng/ml) and NNAL d_3_ (3 ng/ml) were added to 1 ml of urine sample. 100 µL 2 M sodium potassium phosphate buffer pH 7, and 100 µL β-glucuronidase (from E. coli type IXA Sigma-Aldrich, 1000 units) dissolved in 0.1 M phosphate buffer were added to the samples as well. Samples were placed in an incubator overnight at 37°C. (This step hydrolyzes glucuronide conjugates to the parent metabolite. This is done because a large percentage of NNAL is conjugated, (Carmella et al., 2002) and providing results as “total NNAL” improves sensitivity as well as reduces variability due to individual differences in the extent of conjugation. The amount of enzyme added is comparable to the amount previously shown to maximize deconjugation (Jacob et al., 2008)). To each sample 0.1 ml potassium carbonate (50% w/v), and 3 ml 70:30 toluene/1-butanol were added. The tubes were vortexed 5 min, centrifuged at 4,000 g for 5 min, and the aqueous phase frozen by immersion in a dry ice/acetone bath. The organic phase was poured to a new tube containing 0.5 ml 1 M sulfuric acid. The mixture was vortexed, centrifuged, and the upper layer was poured off and discarded after freezing the aqueous layer in dry ice/acetone. The acid phase containing the analytes washed with 2 ml of 1:2 ethyl acetate/toluene by vortexing, centrifuging and placing in a dry ice/acetone bath to freeze the lower aqueous layer. The upper layer was poured off and discarded. The acid layer was made basic with 0.5 ml of 50% (w/v) potassium carbonate and 3 ml 2:1 toluene/ethyl acetate was added. The mixture was vortexed, centrifuged, and placed in a dry ice/acetone bath to freeze the lower aqueous layer. The organic phase poured into a tube containing 100 µL10% hydrochloric acid in methanol (to prevent evaporation of the analytes by converting them to non-volatile salts) before evaporating to dryness. The residues were derivatized by adding 50 µL hexanoic anhydride and catalyst, 10 μL of 50 mg/ml 4-dimethylaminopyridine (DMAP) in toluene and the tubes were capped and heated at 70 °C for 15 min. Saturated aqueous sodium bicarbonate (0.5 ml) and 3 ml of 10% ethyl acetate in pentane were added. The tubes were placed in a dry ice/acetone bath to freeze the lower aqueous layer, and the organic phase was poured into tubes containing 0.5 ml of 1 M sulfuric acid. The tubes were vortexed, centrifuged, and placed in a dry ice/acetone bath to freeze the aqueous layers. The organic layers were poured off and discarded. The acid layers were washed with 3 ml 10% ethyl acetate in pentane by vortexing, centrifuging, freezing the aqueous layers, pouring off and discarding the organic layers. The acid layers were made basic with 0.5 ml of 50% (w/v) potassium carbonate and then extracted with 3 ml of 10% ethyl acetate in pentane by vortexing, centrifuging, freezing the aqueous layer, and pouring organic layer to a new set tubes for evaporation. Evaporation to dryness was carried out using a SpeedVac. The residues were reconstituted in 200 µL 20% methanol in 0.1% formic acid. Standards and QC samples were prepared by spiking pooled non-smokers’ urine with the analytes, spanning the expected concentration ranges. QC sample concentrations were for anabasine, anatabine, and anatalline, in ng/mL: 30, 5, 0.25, 0.1, 0.03, and 0 = blank urine matrix For NNAL they were, in ng/mL: 3, 0.5, 0.025, 0.01, 0.003, and 0 = blank urine matrix. Duplicate standards and QCs were extracted and analyzed with each sample run.

### Chromatography and Mass Spectrometry, Method 2

A 20 µL aliquot of the extract was injected *via* the autosampler into the Vantage LC-MS/MS system. Chromatography was performed on a Phenomenex Kinetex phenyl hexyl 100 A column (2.6 µm particle size, 3 mm × 150 mm, Phenomenex, United States) at 50°C with a flow rate of 0.6 ml/min, applying a gradient consisting of 10 mM ammonium formate in 5% methanol (A) and methanol (B). Preparation of 1 L of mobile phase A involves mixing 0.625 g of ammonium formate, 50 ml methanol and HPLC grade water to volume. Gradient conditions were as follows: 0 min: 45% A, 0–3 min: 45% A, 3–4.5 min: 45–0% A, 4.5–5 min: 0% A, 5–5.5 min: 0–45% A, 5.5–8 min: 45% A. Positive electrospray ionization (ESI) was used. The spray voltage was 3500, the vaporizer temperature was 440°C, the capillary temperature was 395°C, the sheath gas Pressure was 45 psi, the auxiliary gas pressure was 5 psi, and the ion sweep gas pressure was 0 psi. The resolution of the first quadrupole, FWHM, was set at 0.5 amu, the resolution of the third quadrupole was set at 0.7 amu FWHM. The MS/MS system was run in the selected reaction monitoring (SRM) mode. Mass transitions for the analytes and internal standards are in Table 1.

### Data Analysis Method 2

Calibration for quantitation was carried out as described for Method 1 above. Typical equations and correlation coefficients for representative standard curves are in the Supplementary Material document.

### Methods Validation

Precision, accuracy, and limits of quantitation were determined by replicate analysis of spiked urine samples, at concentrations spanning the expected concentration ranges (Tables 2, 3) as described by Shah *et al.* (Shah et al., 2000) and Viswanathan *et al.*(Viswanathan et al., 2007) Briefly, the criteria are that the precision should be RSD less than 15%, except at the LOQ which should be less than 20%. The accuracy should be within ± 15% of the expected amount except at the LOQ in which ± 20% is acceptable. The LOQ was the lowest concentration meeting these criteria. Lack of carryover was verified by analysis of analytical blanks, extracts of non-smokers’ urine described above. Blanks also served to identify potentially interfering substances derived from the sample matrix or from reagents and solvents used in extractions.

**TABLE 2:** Method 1 precision and accuracy for determination of anabasine, anatabine, anatalline, anatalline metabolite, and nicotelline in Urine. 6 replicate analyses.^1^

Analyte	Added amount (ng/ml)	Measured mean (ng/ml)	Accuracy (percent of expected)	Precision CV (%)
Anabasine	30.0	30.1	102	1.1
LLOQ = 0.1 ng/ml	5.00	5.58	112	1.7
	0.250	0.225	90	10.2
	0.100	0.087	87	9.4
Anatabine	30.0	28.6	95	2.6
LLOQ = 0.1 ng/ml	5.00	5.28	106	1.9
	0.250	0.254	102	2.1
	0.100	0.101	101	2.4
Anatalline	30.0	28.1	94	6.5
LLOQ = 0.1 ng/ml	5.00	5.36	107	5.8
	0.250	0.243	97	5.4
	0.100	0.080	80	2.2
Anatalline Metabolite	3.00	3.25	108	2.4
LLOQ = 0.5 ng/ml	0.500	0.541	108	1.7
Nicotelline	3.00	2.73	91	1.8
LLOQ = 0.01 ng/ml	0.500	0.460	92	2.0
	0.025	0.022	89	7.5
	0.010	0.0090	90	3.5

1 Pooled non-smokers’ urine was spiked with analytes to the specified concentrations. LLOQ = Lower Limit of Quantitation. Individual sample data are in the Supplementary Material document.

**TABLE 3:** Method 2 precision and accuracy for determination of anabasine, anatabine, anatalline, and NNAL in Urine. 6 replicate analyses.^1^

Analyte	Added amount (ng/ml)	Measured mean (ng/ml)	Accuracy (percent of expected)	Precision CV (%)
Anabasine	30.0	30.3	101	3.7
LLOQ = 0.030 ng/ml	5.00	5.16	103	1.5
	0.250	0.260	104	2.7
	0.100	0.106	106	6.7
	0.030	0.027	91.5	8.7
	Smoker’s Urine	5.35	NA	2.5
Anatabine	30.0	30.8	103	2.4
LLOQ = 0.030 ng/ml	5.00	4.79	95.9	2.5
	0.250	0.257	103	2.1
	0.100	0.105	105	3.3
	0.030	0.029	96.5	4.7
	Smoker’s Urine	4.80	NA	1.1
Anatalline	30.0	34.3	114	2.7
LLOQ = 0.030 ng/ml	5.00	5.62	112	3.3
	0.250	0.270	108	4.8
	0.100	0.108	108	5.5
	0.030	0.033	110	7.9
	Smoker’s Urine	2.77	NA	0.9
NNAL	3.00	3.25	108	2.4
LLOQ = 0.0030 ng/ml	0.500	0.541	108	1.7
	0.025	0.028	113	3.2
	0.010	0.010	103	5.2
	0.003	0.0033	109	5.1
	Smoker’s Urine	0.0479	NA	3.6

1 Pooled non-smokers’ urine was spiked with analytes to the specified concentrations. LLOQ = Lower Limit of Quantitation. Individual sample data are in the Supplementary Material document.

### Human Urine Samples

Urine samples were available from previous studies. (Benowitz et al., 2012; St Helen et al., 2020b) All studies received the approval of the appropriate institutional review boards. Twenty urine samples from cigarette smokers were obtained at baseline in a longitudinal study of progressive reduction in the nicotine concentrations of cigarettes. (Benowitz et al., 2012) Nineteen urine samples from non-smokers not exposed to SHS were obtained in San Francisco. Smoking status and SHS exposure was by self-report and/or the nicotine metabolite cotinine concentration below the established cutpoint of 40 ng/ml for distinguishing smokers from non-smokers. (Edwards et al., 2021) Urine samples from 36 dual users of combusted cigarettes and e-cigarettes were 24 h collections in a crossover study of use of e-cigarettes and combusted cigarettes carried out on the Clinical Research Center at Zuckerberg San Francisco General Hospital. (St Helen et al., 2020b) Urine samples were collected during 2 days of *ad libitum* vaping or cigarette smoking and 2 days of enforced abstinence.

## Results and Discussion

Two methods for minor tobacco alkaloids were developed, with the goal of simultaneously measuring urine concentrations of multiple analytes of interest. These include the established biomarkers anabasine and anatabine, and nicotelline, that we proposed as a biomarker for the particulate matter derived from tobacco smoke, (Jacob et al., 2013) anatalline, a little-studied tobacco alkaloid that we are developing as a new biomarker, and NNAL, a well-established biomarker for the tobacco-specific nitrosamine NNK (Hecht, 2002).

Method 1 is based on an LC-MS/MS method we developed for nicotelline. Since little if any nicotelline is excreted in urine unchanged, and the only metabolites characterized so far are N-oxides, the method involves treating urine with titanium trichloride, which reduces the N-oxides back to nicotelline that can be readily quantitated. (Jacob et al., 2013) (Figure 3). We have modified this method to include anabasine, anatabine, anatalline, and a lactam metabolite of anatalline as analytes. Nicotelline is highly selective for tobacco, and was undetectable or present at very low concentrations in 70 e-liquids that we analyzed. (Jacob et al., 2020) Therefore, we proposed that nicotelline could be used as a biomarker for combusted tobacco use in people using e-cigarettes. (Jacob et al., 2020) Nicotelline has a short half-life, 2–3 h, and is useful for detecting recent tobacco use. (Jacob et al., 2013) But nicotelline concentrations are undetectable in 12–24 h after tobacco use ceases. The tobacco-specific nitrosamine metabolite NNAL has also been used as a biomarker for tobacco use in ENDS users, but NNAL has a very long half-life, >10 days (Hecht et al., 1999; Goniewicz et al., 2009) and it can be measured in urine for several weeks after tobacco cessation. Consequently, biomarkers with half-lives longer than nicotelline, but shorter than NNAL, such as anabasine, anatabine, and anatalline would be also useful in studies of the short-term effects of switching from combusted cigarettes to e-cigarettes.

The minor alkaloids anabasine and anatabine have been used as biomarkers for tobacco use in people using nicotine-containing medications for tobacco cessation. (Jacob et al., 2002; Suh-Lailam et al., 2014; Sinclair et al., 2020) They have also been used as biomarkers for tobacco use in people using ENDS. (Berlin et al., 2019) Anabasine and anatabine have half-lives of about 16 and 10 h, respectively, and can detect tobacco use for a few days following tobacco cessation. (Jacob et al., 1999) Therefore, they are complementary to nicotelline (t ½ = 2 h) and NNAL (t ½ > 10 days). (Benowitz et al., 2020) However, in contrast to nicotelline, anabasine and anatabine have been found in e-liquids, sometimes in concentrations as high as in cigarette tobacco normalized to nicotine, (Palazzolo et al., 2019; Jacob et al., 2020) which may limit their utility as biomarkers selective for tobacco use. Anatalline is another minor alkaloid that we are developing as a biomarker. Like nicotelline, (Jacob et al., 2020) and in contrast to anabasine and anatabine, anatalline was undetectable or present at very low concentrations in 70 e-liquids that we analyzed. (Table 4). Interestingly, nicotelline does not appear to be naturally occurring (probably not biosynthesized) in the tobacco plant, and is mainly formed from anatalline by pyrolysis and oxidation in burning tobacco. This was demonstrated by adding anatalline to a non-tobacco plant material, oregano, preparing a “cigarette” from this, combusting, collecting and analyzing the smoke. In parallel, an oregano “cigarette” without anatalline was prepared and combusted. Nicotelline was detected in the smoke from the oregano “cigarette” spiked with anatalline, but not in the smoke from the “cigarette” without anatalline. (Jacob et al., 2013) This demonstrated that nicotelline can be formed from anatalline during combustion conditions, and that neither nicotelline nor anatalline are likely to be formed by combustion/pyrolysis of organic materials. The half-life of anatalline *appears* to be similar to that of anabasine or somewhat longer, which is apparent from the data presented in Figure 8, but additional studies will be required to determine its half-life. Therefore, we propose that anatalline would be a more selective biomarker than anabasine and anatabine for tobacco use in ENDS users, but otherwise would have similar attributes.

**TABLE 4:** Mean concentrations of nicotelline, anatalline, anabasine and anatabine, normalized to nicotine, in 70 e-liquids compared to the corresponding concentrations in a mainstream smoke of a reference cigarette. BLQ = Below the limit of quantitation.^1^ Concentrations of nicotine and the other alkaloids were determined by the method of Jacob *et al.*, described in reference (Jacob et al., 2020). The 70 e-liquids are also described in Supplementary Information for reference (Jacob et al., 2020).

Product	N	Mean concentration (range) µg/mg nicotine			
		Nicotelline	Anatalline	Anabasine	Anatabine
E-Liquids	70	0.00016 (BLQ—0.0043) 91% BLQ	0.0042 (BLQ—0.081) 86% BLQ	0.41 (BLQ—2.80) 40% BLQ	1.00 (BLQ—8.89) 7% BLQ
Mainstream Smoke, 1R6F Reference Cigarette, HCI Regimen		1.55	1.92	1.71	6.14

^1^LOQs were: nicotine, 1 μg/ml; nicotelline, 0.15 ng/ml; anatalline, 0.46 ng/ml; anabasine, 4.1 ng/ml; anatabine, 0.05 ng/ml.

**FIGURE 5:**
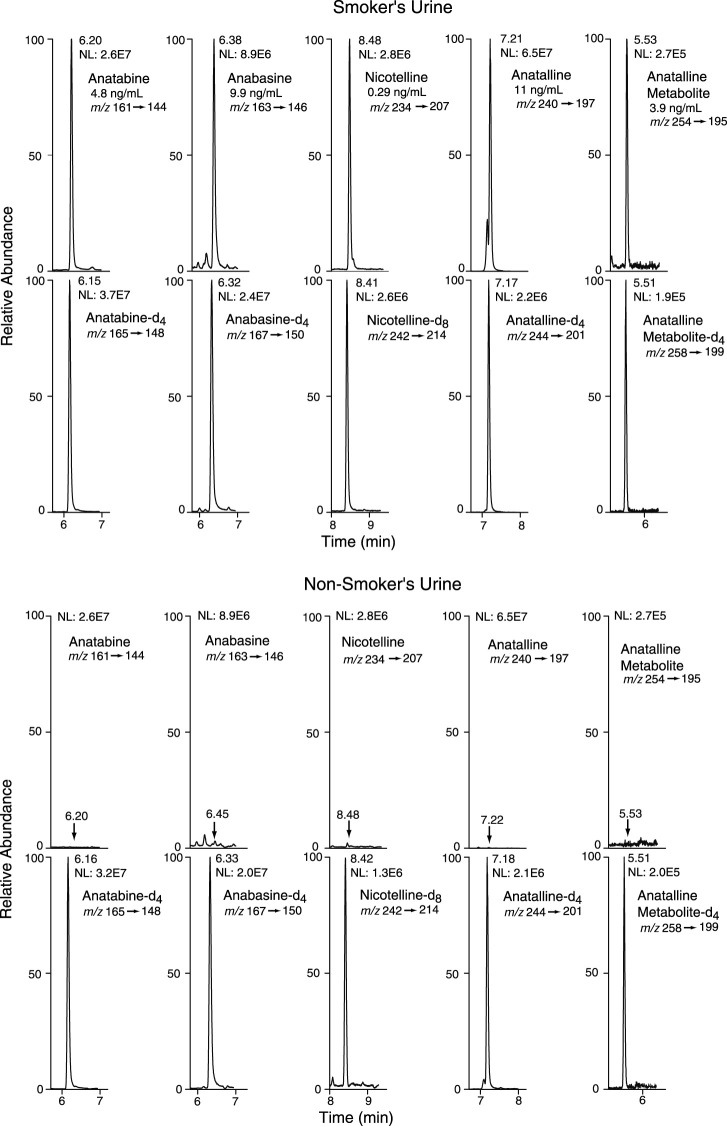
Selected reaction monitoring (SRM) chromatograms from urine analyzed by Method 1. Analyte peaks in the non-smoker’s chromatogram were scaled to match those of the smoker’s urine chromatogram. The internal standard concentrations were anabasine-d_4_ and anatabine d_4_, 20 ng/ml; anatalline-d_4_, 0.8 ng/ml; anatalline metabolite-d_4_, 30 ng/ml; nicotelline-d_8_, 2 ng/ml.

Method 1 uses the same sample prep as our published LC-MS/MS method for nicotelline. (Jacob et al., 2013) Chromatography and mass spectrometry parameters were modified to include anabasine, anatabine and anatalline. Data on precision, accuracy, and limits of quantitation for the method are presented in Table 2. Representative selected reaction monitoring (SRM) chromatograms are in Figure 5. This method was used to obtain data on concentrations of anabasine, anatabine, anatalline, an anatalline metabolite, and nicotelline in cigarette smokers and in non-smokers urine, summarized in Table 5. Concentrations of the widely used biomarkers for nicotine exposure, cotinine and *trans*-3’-hydroxycotinine are included for comparison, since concentrations, especially cotinine, have been used for many years as biomarkers of tobacco exposure, and can serve as an index of the extent of tobacco and/or nicotine product use. Applications of the minor alkaloids in studies of dual use of ENDS and conventional tobacco products would likely include cotinine concentrations as well to assess overall nicotine product use. We believe that this is the first published data on concentrations of anatalline and its metabolite in human urine.

**TABLE 5:** Concentrations of biomarkers in urine of 20 cigarette smokers and 19 non-smokers^1^.

Biomarker	Smokers^2^ ^,^ ^3^	Non-smokers^4^
Anabasine, ng/mL		
Mean	14.1	0.346
Range	0.503–47.2	BLQ—5.23
SD	13.1	1.2
Detection Frequency (LLOQ = 0.100)	100%	32%
Anatabine, ng/mL		
Mean	11.7	BLQ
Range	0.508–33.7	BLQ
SD	10.5	
Detection Frequency (LLOQ = 0.100)	100%	0%
Anatalline, ng/mL		
Mean	14.3	BLQ
Range	0.595–78.1	BLQ
SD	17.4	
Detection Frequency (LLOQ = 0.050)	100%	0%
Anatalline Metabolite, ng/mL		
Mean	3.15	BLQ
Range	BLQ—9.07	BLQ
SD	2.5	
Detection Frequency (LLOQ = 0.500)	95%	0%
Nicotelline, ng/mL		
Mean	1.70	BLQ
Range	0.095–6.43	BLQ—0.059
SD	1.7	17
Detection Frequency (LLOQ = 0.010)	100%	26%
Cotinine, ng/mL		
Mean	1,557	0.20
Range	501–3,245	BLQ—2.6
SD	763	0.59
Detection Frequency (LLOQ = 10, 0.05)^5^	100%	58%
3’-Hydroxycotinine, ng/mL		
Mean	6,458	0.88
Range	376–12,547	BLQ—9.8
SD	3,580	2.2
Detection Frequency (LLOQs = 10, 0.1)^5^	100%	84%

1 Concentrations of anabasine, anatabine, anatalline, anatalline metabolite, and nicotelline were determined by Method 1. Concentrations of cotinine and 3’-hydroxycotinine were determined by the methods of Jacob *et al.* [Reference (Jacob et al., 2011)]. Concentrations of NNAL were determined by the method of Jacob *et al.* [Reference (Jacob et al., 2008)]. Individual sample data are in the Supplementary Material document.

2 Smokers smoked an average of 18.9 cigarettes per day, 95% confidence interval = 15.5–22.3

3 If below the limit of quantitation (BLQ), LLOQ/square root 2 was used.

4 If below the limit of quantitation (BLQ), 0 was used.

5 Two method variations were used. LLOQ is 10 for smokers’ urine and lower for non-smokers’ urine

BLQ = Below the limit of quantitation

**FIGURE 6:**
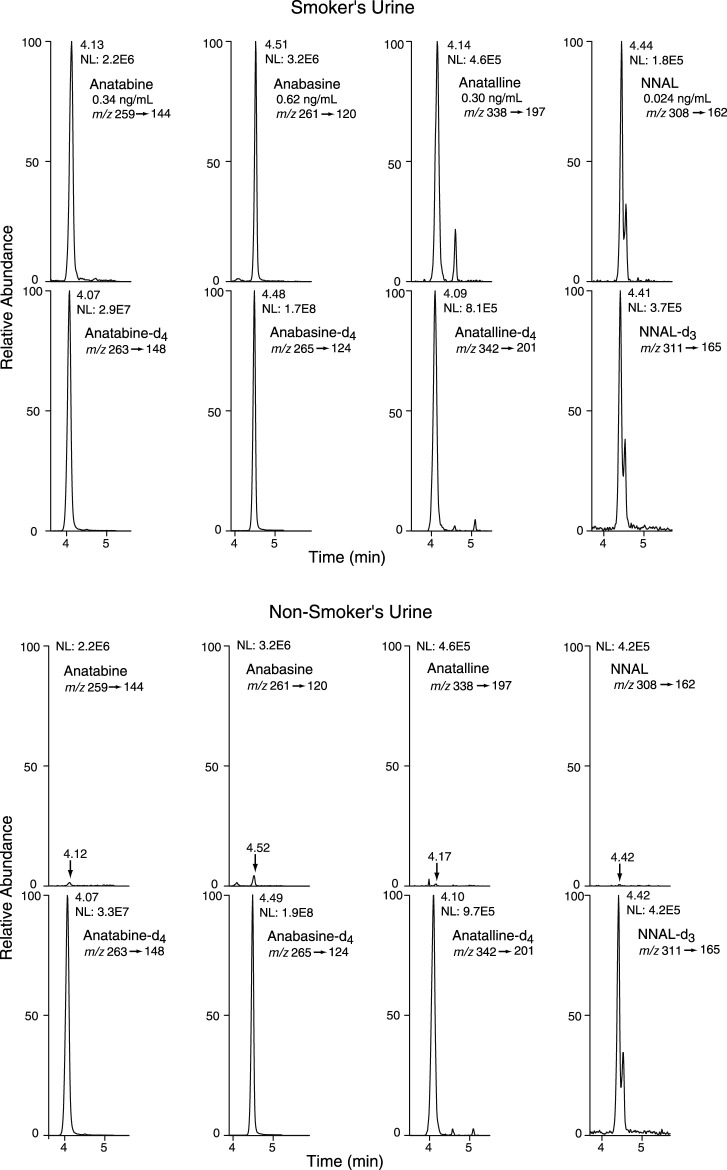
Selected reaction monitoring (SRM) chromatograms from urine analyzed by Method 2. Analyte peaks in the non-smoker’s chromatogram were scaled to match those of the smoker’s urine chromatogram. The internal standard concentrations were anabasine-d_4_ and anatabine d_4_, 20 ng/ml; anatalline-d_4_, 0.8 ng/ml; NNAL-d_3_, 0.6 ng/ml. There are two partially resolved NNAL and NNAL-d_3_ peaks, because NNAL exists as two slowly (on the timescale of the chromatography) equilibrating *syn* and *anti* isomers with respect to the N-N bond.

**FIGURE 7:**
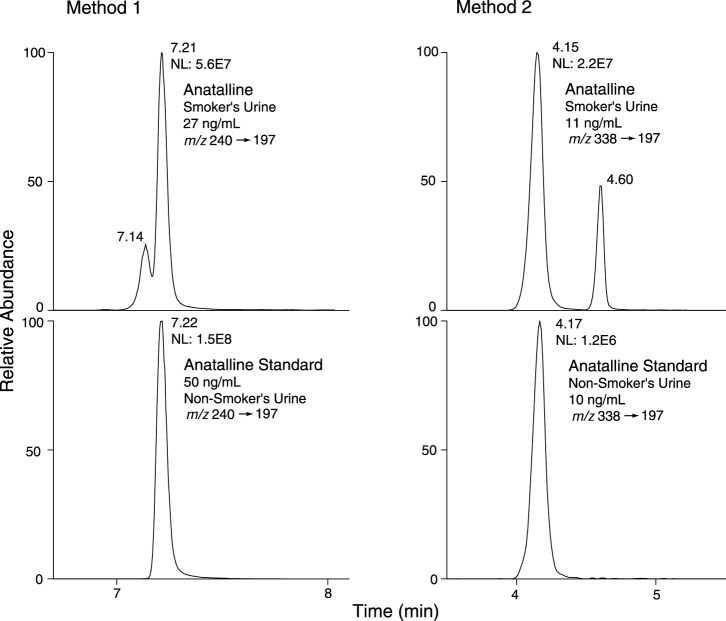
Selected reaction monitoring (SRM) chromatograms from smokers’ urine and non-smokers’ urine spiked with anatalline standard, analyzed by Method 1 and 2. Detection of a possible isomer of anatalline.

Method 2 is based on a LC-MS/MS method we developed for the tobacco-specific nitrosamine metabolite NNAL. (Jacob et al., 2008) It involves treating extracts with hexanoic anhydride to give an ester derivative, that facilitates sample clean up *via* extraction with non-polar solvents, resulting in increased sensitivity compared to analyses with underivatized NNAL. Hexanoic andydride converts anabasine, anatabine and anatalline to hexanoic acid amides that likewise facilitates clean up of extracts and allows simultaneous determination along with NNAL. (Figure 4) Data on precision, accuracy, and limits of quantitation for the method are presented in Table 3. Representative selected reaction monitoring (SRM) chromatograms are in Figure 6. We think that it is interesting to note that in all SRM chromatograms from smokers’ urine extracts a peak with a retention time of about 0.45 min longer than the anatalline peak is observed, not found in chromatograms from non-smokers urine spiked with the anatalline standard. In Method 1 chromatograms, SRM chromatograms from smokers’ urine extracts a partially resolved peak with a shorter retention time than the anatalline is observed, not found in chromatograms from non-smokers spiked with the anatalline standard. (Figures 5–7). These peaks are clearly derived from a substance inhaled in cigarette smoke, possibly an isomeric alkaloid, and chromatographic separation is needed to accurately quantify anatalline. We also observed a peak partially resolved from anatalline in SRM chromatograms from a cigarette tobacco extract. (Jacob et al., 2013)

**FIGURE 8:**
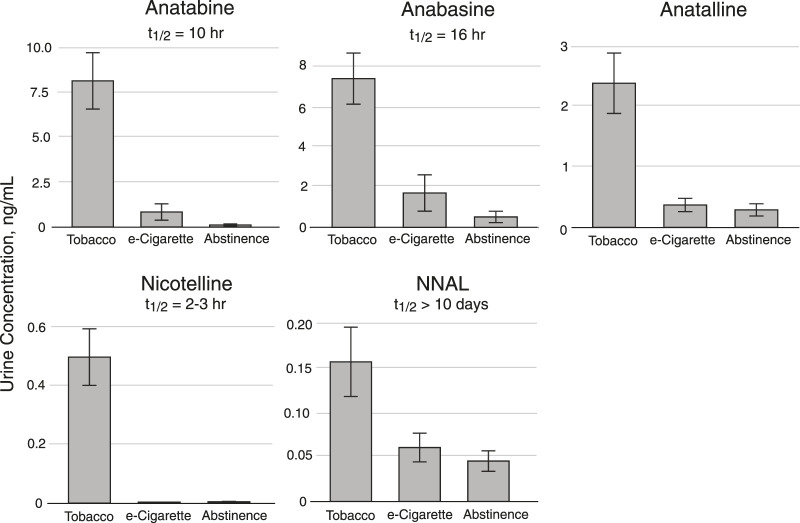
Urine concentrations of Anatabine, anabasine, anatalline, nicotelline, and NNAL in dual users of combusted cigarettes and e-cigarettes enrolled in a crossover study carried out on a research ward. Participants were inpatients in three 2-day study blocks. For this subset of participants (*n* = 19), the 2-day abstinence block immediately followed the e-cigarette block. For the five days prior to the e-cigarette block, participants were instructed to use their usual e-cigarette product *ad libitum* and not use other tobacco or nicotine-containing products. However, since they were outpatients, compliance could not be enforced. Urine (24 h pool) collected during the second day of each 2-day study block was analyzed for the biomarkers. The “Tobacco” columns data were obtained during the 2-day cigarette smoking block. Individual sample data are in the Supplementary Material document.

Method 2 has been used in a crossover study of dual users of e-cigarettes and combusted cigarettes. (St Helen et al., 2020b) The participants used e-cigarettes or smoked combusted cigarettes in separate 2-day study blocks, followed by a third 2-day block when they abstained from the use of any nicotine product, enforced by the study being carried out on a research ward with no access to nicotine-containing products. Urine samples were collected and analyzed for biomarker concentrations (Figure 8). These data are from a subset of participants (*n* = 19) in which the 2-day abstinence block immediately followed the e-cigarette block. For the five days prior to the e-cigarette block, participants were instructed to use their usual e-cigarette product and not use other tobacco or nicotine-containing products. However, since they were outpatients, compliance could not be enforced. We used data from this study, in which concentrations of anabasine, anatabine, anatalline, and NNAL were determined using Method 2, and nicotelline was available from previous analyses using a published method, (Jacob et al., 2013) to illustrate the attributes of the various biomarkers in terms of their different rates of elimination (Figure 8). Nicotelline, with a half-life of 2–3 h detects recent combusted tobacco use, and concentrations were near or below the limit of quantitation (LOQ) during the inpatient e-cigarette and abstinence blocks. Anatabine (t_1/2_ = 10 h) was measurable during the e-cigarette block but not the abstinence block. Anabasine, anatalline, and NNAL were measurable in both the e-cigarette and abstinence blocks because their half-lives are too long to fall below the LOQ during the course of this study. (Benowitz et al., 2020) NNAL, due to its very long (>10 days) half-life can be detected for several weeks following tobacco cessation. Depending on the goals of a particular study, confirmation of short-term or long-term tobacco cessation may be desirable. NNAL would be the most useful for population studies in which any tobacco use in the past 2–3 months is of interest. The minor alkaloids are more relevant for assessment of short-term cigarette smoking, such as looking for point prevalence of smoking in smoking cessation trials. Also, the high sensitivity of Method 2 extends the time frame of applicability of anabasine and anatabine in which these biomarkers can be measured following tobacco cessation, and makes low-level exposure assessment possible. The lower limits of quantitation for anabasine and anatabine in Method 2 are 0.03 ng/ml. For comparison, the LLOQs reported in Wei *et al.* for a method used in large, population-scale studies including the National Health and Nutrition Examination Survey (NHANES) and the Population Assessment of Tobacco and Health (PATH) study are an order of magnitude higher, 0.5 and 0.4 ng/ml, respectively. (Wei et al., 2014) The LLOQ of NNAL in Method 2, 0.003 ng/ml, is sufficient for measuring exposure in cigarette and cigar smokers and smokeless tobacco users, since this is well below the cutpoint of 0.010–0.040 ng/ml for distinguishing active use from passive exposure. (Benowitz et al., 2020) For low-levels of secondhand smoke exposure, a more sensitive method may be advantageous, such as the method we reported that uses a larger urine volume but the same sample prep as Method 2, and has a LLOQ of 0.00025 ng/ml. (Jacob et al., 2008)

## Summary and Conclusion

The goal of our studies was to develop methods to simultaneously quantify multiple biomarkers useful in studies of dual use of ENDS and combusted cigarettes.

Both of the methods we describe include anabasine, anatabine, and anatalline as analytes. Anabasine and anatabine have been used for a number of years as biomarkers to distinguish the use of nicotine-containing medications from the use of conventional tobacco products, and continue to be used in large population studies, such as the National Health and Nutrition Examination Survey (NHANES) and the Population Assessment of Tobacco and Health (PATH) study. (Wei et al., 2014) They have also been used to distinguish e-cigarette use from combusted cigarette use. (Berlin et al., 2019) In this report, we introduce anatalline as a new biomarker, with a rate of elimination similar to anabasine and anatabine, based on preliminary data, as illustrated in Figure 8. We suggest that anatalline has similar attributes to anabasine and anatabine, but unlike those two biomarkers it has not been found to any significant extent in e-liquids (Table 4 **)** and therefore should be more selective for the use of conventional tobacco products.

The two methods differ in that Method 1 also measures nicotelline, and Method 2 also measures NNAL. Method 1 cannot measure NNAL because the titanium trichloride reagent used to reduce nicotelline N-oxides decomposes NNAL. Method 2 uses derivatization with hexanoic anhydride to enhance sensitivity for NNAL and also enhances the sensitivity for anabasine, anatabine, and anatalline. Nicotelline cannot be measured with Method 2, because little if any is excreted unchanged, and the only known metabolites are N-oxides, and reduction of these with titanium trichloride to nicotelline is required for sensitive quantitation. (Jacob et al., 2013) Another difference between the methods is that Method 2 employs a deconjugation step using β-glucuronidase, since about 50% of NNAL is excreted as glucronides. Since nicotine and cotinine are N-conjugated (pyridine nitrogen), the possibility exists that anabasine, anatabine, and anatalline might likewise be N-conjugated. To address this possibility, we analyzed a pooled smokers’ urine with and without deconjugation using Method 2. The data are presented in Table 6. Anabasine and anatabine were excreted as glucuronides to the extent of 20 and 31%, respectively, but interestingly glucuronidation of anatalline was not detected. As expected, NNAL was about 50% conjugated.

**TABLE 6:** Concentrations of anabasine, anatabine, anatalline, and NNAL in pooled smokers’ urine, with and without deconjugation using β-glucuronidase. Mean of 6 replicate analyses using Method 2.^1^

Analyte	Total (Enzyme deconjugated) ng/mL (SD)	Free (No deconjugation) ng/mL (SD)	Percent conjugated (%)
Anabasine	5.9 (0.13)	4.7 (0.13)	20
Anatabine	5.5 (0.18)	3.8 (0.14)	31
Anatalline	2.9 (0.11)	2.9 (0.12)	0
NNAL	0.087 (0.0020)	0.044 (0.0013)	49

1 Individual sample data are in the Supplementary Material document.

Method 1 also measures a lactam metabolite (Figure 1) of anatalline, which is reported for the first time. The lactam metabolite of nicotine, cotinine is a valuable biomarker for various reasons, including a longer half-life than nicotine. (Benowitz et al., 2020) By analogy, the lactam metabolite of anatalline might likewise be a useful biomarker. Consequently, we postulated the existence of this metabolite, and found that it indeed it is excreted in urine of smokers (Table 5). But, it proved to be a difficult analyte, perhaps due to its polarity and inefficient extraction limiting method sensitivity. This is reflected in the relatively high LOQ (0.5 ng/ml compared to the other analytes and the correlation coefficient of the standard curve. However, due to its potential attributes, further studies of this metabolite including efforts to develop a more sensitive method may be warranted.

Concerning the relative merits of these two new methods, the choice may depend on which analytes are most important for a particular study. Method 1 simultaneously measures nicotelline, anabasine, anatabine, and anatalline. Method 1 would be most appropriate if a measure of recent cigarette smoking, within 24 h, was desired, which would be provided by nicotelline, with a half-life of 2–3 h. This method also detects smoking occurring over the past several days, from anatabine, anabasine, and anatalline, with half-lives ranging from 10 h to 16 or more hours. Anabasine, anatabine, and anatalline may be useful for confirming tobacco cessation over this time frame, such as studies of the effects of short-term switching from combusted cigarettes to e-cigarettes. Method 2 simultaneously measures anabasine, anatabine, anatalline, and NNAL. Method 2 would be most appropriate if the goal were to detect, and measure the extent of smoking occurring over several weeks, which would be provided by NNAL, which has a half-life in excess of 10 days. Method 2 will also detect smoking occurring within several days, from concentrations of anabasine, anatabine, and anatalline. Method 2 would also be most appropriate for low-level exposure assessment, because it is more sensitive than Method 1 by a factor of about 3 for anabasine, anatabine, anatalline (Tables 2, 3).

The value of multiple tobacco biomarkers with a range of elimination rates is discussed in a recent publication, including a figure illustrating the time course for concentrations to fall below the LLOQ. (Benowitz et al., 2020)

In conclusion, two new methods for tobacco biomarkers have been developed, that can be applied to studies of dual users of ENDS and conventional tobacco products. Advantages include simultaneous determination of multiple analytes, and improved sensitivity compared to previous methods that could be useful for low-level exposures. We also introduce a new biomarker, anatalline. Anatalline has similar attributes to anabasine and anatabine, and may have greater specificity for tobacco than those two alkaloids.

## Data Availability

The original contributions presented in the study are included in the article/Supplementary Material, further inquiries can be directed to the corresponding author.
