# Supplementary material for: Minor Tobacco Alkaloids as Biomarkers to Distinguish Combusted Tobacco Use From Electronic Nicotine Delivery Systems Use. Two New Analytical Methods
Source: Front Chem. 2022 Jun 1;10:749089. doi: 10.3389/fchem.2022.749089 (PMC9198481; doi:10.3389/fchem.2022.749089)
Supplement: Supplementary file 1 [file DataSheet1.PDF]

Supplementary Material

Supplementary Data

1. Data for Calibration Curves

2. Table 2 Data. Precision and Accuracy, Method 1

3. Table 3 Data. Precision and Accuracy, Method 2

4. Table 5 Data. Concentrations of Alkaloids and Nicotine Metabolites in Urine of Smokers and Non-Smokers

5. Table 6 Data. Deconjugation Data

6. Figure 8 Data. Alkaloid and NNAL Concentrations

1. Data for Typical Calibration Curves

Method	Analyte	Concentration Range	Equation and Correlation Coefficient
1	Anabasine	0.020 – 50 ng/mL	Y = 0.0300399+0.000103489*X R^2 = 0.9977
1	Anatabine	0.020 – 50 ng/mL	Y = 0.000595127+7.57252e-005*X R^2 = 0.9992
1	Anatalline	0.020 – 50 ng/mL	Y = 0.0325992+0.0039389*X R^2 = 0.9558
1	Anatalline Metabolite	0.0020 – 5 ng/mL	Y = 0.00256367+0.000144511*X R^2 = 0.9270
1	Nicotelline	0.0020 – 5 ng/mL	Y = 0.00125612+0.000172009*X R^2 = 0.9962
2	Anabasine	0.020 – 50 ng/mL	Y = 0.000716607+2.6783e-005*X R^2 = 0.9981
2	Anatabine	0.020 – 50 ng/mL	Y = 0.000622128+0.000218806*X R^2 = 0.9812
2	Anatalline	0.020 – 50 ng/mL	Y = 0.00222021+0.00190516*X R^2 = 0.9989
2	NNAL	0.0020 – 5 ng/mL	Y = 0.00471107+0.0172639*X R^2 = 0.9995

2. Table 2 Data

Anabasine Precision and Accuracy, Method 1

Sample	Spiked Urine pg/mL	Low-Level Standard Curve Calculated Amount pg/mL	High-Level Standard Curve Calculated Amount pg/mL	Mean	Std Dev	CV	Accuracy Percent of Expected
QA	**30000**	25469.0	30653.3	**30626.8**	324.2	**1.1**	**102%**
QA		25690.7	30920.3				
QA		25145.5	30263.7				
QA		25402.8	30573.6				
QA		25808.1	31061.8				
QA		25165.9	30288.3				
QB	**5000**	4740.1	5446.5	**5578.9**	92.6	**1.7**	**112%**
QB		4940.1	5715.2				
QB		4858.9	5598.6				
QB		4794.6	5625.5				
QB		4773.2	5573.4				
QB		4722.9	5514.3				
QC	**250**	216.1	236.9	**225.3**	22.9	**10.2**	**90%**
QC		184.5	198.8				
QC		225.3	248.0				
QC		246.0	272.9				
QC		243.9	270.4				
QC		235.8	260.6				
QE	**100**	96.5	92.8	**87.2**	8.2	**9.4**	**87%**
QE		83.7	77.4				
QE		81.3	74.5				

**Low Curve Cutoff**	**500**
**LOQ**	**100**

Anatabine Precision and Accuracy, Method 1

Sample	Spiked Urine pg/mL	Low-Level Standard Curve Calculated Amount pg/mL	High-Level Standard Curve Calculated Amount pg/mL	Mean	Std Dev	CV	Accuracy Percent of Expected
QA	**30000**	26794.5	27221.8	**28598.8**	752.1	**2.6**	**95%**
QA		27101.0	29342.4				
QA		26814.9	29032.6				
QA		26491.2	28682.1				
QA		26743.1	28954.9				
QA		26192.9	28359.1				
QB	**5000**	4996.4	5095.8	**5276.8**	102.5	**1.9**	**106%**
QB		5357.7	5408.4				
QB		5188.4	5315.8				
QB		5191.4	5302.3				
QB		5103.4	5258.8				
QB		5182.6	5279.5				
QC	**250**	247.0	260.5	**253.9**	5.3	**2.1**	**102%**
QC		262.4	277.2				
QC		250.6	264.5				
QC		256.2	270.5				
QC		252.5	266.5				
QC		254.8	269.0				
QE	**100**	103.4	105.0	**100.8**	2.4	**2.4**	**101%**
QE		100.3	101.7				
QE		98.6	99.9				

**Low Curve Cutoff**	**500**
**LOQ**	**100**

Anatalline Precision and Accuracy, Method 1

Sample	Spiked Urine pg/mL	Calculated Amount pg/mL	Mean	Std Dev	CV	Accuracy Percent of Expected
QA	**30000**	29008.9	**28141.7**	1839.4	**6.5**	**94%**
QA		31434.8				
QA		27875.5				
QA		27130.5				
QA		26510.1				
QA		26890.6				
QB	**5000**	5423.6	**5355.1**	310.4	**5.8**	**107%**
QB		5703.5				
QB		5727.0				
QB		5111.8				
QB		5013.8				
QB		5151.0				
QC	**250**	260.9	**243.0**	13.1	**5.4**	**97%**
QC		257.8				
QC		237.3				
QC		239.5				
QC		232.2				
QC		230.2				
QE	**100**	81.2	**79.7**	1.8	**2.2**	**80%**
QE		77.7				
QE		80.1				

**LOQ**	**100**

Anatalline Metabolite Precision and Accuracy, Method 1

Sample	Spiked Urine pg/mL	Calculated Amount pg/mL	Mean	Std Dev	CV	Accuracy Percent of Expected
QA	**30000**	2786.5	**2582.7**	197.3	**7.6**	**86%**
QA		2753.5				
QA		2683.8				
QA		2523.6				
QA		2482.7				
QA		2266.2				
QB	**500**	476.9	**452.4**	57.6	**12.7**	**90%**
QB		547.8				
QB		455.1				
QB		398.5				
QB		389.5				
QB		446.4				

**LOQ**	**500**

Nicotelline Precision and Accuracy, Method 1

Sample	Spiked Urine pg/mL	Calculated Amount pg/mL	Mean	Std Dev	CV	Accuracy Percent of Expected
QA	**3000**	2709.9	**2726.9**	49.7	**1.8**	**91%**
QA		2724.6				
QA		2801.7				
QA		2699.7				
QA		2764.4				
QA		2661.0				
QB	**500**	452.5	**459.8**	9.0	**2.0**	**92%**
QB		466.7				
QB		458.7				
QB		462.5				
QB		447.0				
QB		471.3				
QC	**25**	20.5	**22.2**	1.7	**7.5**	**89%**
QC		25.2				
QC		21.7				
QC		22.6				
QC		21.0				
QC		22.2				
QE	**10**	9.1	**9.0**	0.3	**3.5**	**90%**
QE		8.7				
QE		9.3				

**LOQ**	**10**

3. Table 3 Data
Method 2 Precision and Accuracy

calibrated with full standard curve

QC A (30K/3K)	anabasine pg/mL	anatabine pg/mL	anatalline pg/mL	NNAL pg/mL
qc_A	30690.89	31384.67	34830.30	3139.53
qc_A	28960.53	30213.59	33881.39	3329.59
qc_A	32019.70	30925.22	35156.01	3260.91
qc_A	29232.94	31350.67	34852.99	3289.76
qc_A	30605.00	29509.77	34235.82	3166.39
qc_A	30079.40	31132.51	32589.61	3309.17
**average**	**30264.74**	**30752.74**	**34257.69**	**3249.23**
**std dev**	**1111.50**	**743.84**	**939.55**	**78.39**
**% CV**	**3.7%**	**2.4%**	**2.7%**	**2.4%**
**% accuracy**	**100.9%**	**102.5%**	**114.2%**	**108.3%**

calibrated with full standard curve

QC B (5k/500)	anabasine pg/mL	anatabine pg/mL	anatalline pg/mL	NNAL pg/mL
qc_B	5254.67	4930.85	5860.68	546.07
qc_B	5214.09	4887.61	5494.52	546.17
qc_B	5198.27	4629.18	5695.37	525.27
qc_B	5029.37	4800.41	5730.33	538.13
qc_B	5156.17	4668.01	5344.61	537.95
qc_B	5126.20	4843.75	5595.91	552.21
**average**	**5163.13**	**4793.30**	**5620.24**	**540.97**
**std dev**	**79.39**	**120.87**	**183.27**	**9.41**
**% CV**	**1.5%**	**2.5%**	**3.3%**	**1.7%**
**% accuracy**	**103.3%**	**95.9%**	**112.4%**	**108.2%**

calibrated with low level standard curve

QC C (250/25)	anabasine pg/mL	anatabine pg/mL	anatalline pg/mL	NNAL pg/mL
qc_C	276.96	260.73	256.06	28.62
qc_C	271.39	259.24	273.51	26.52
qc_C	267.06	247.32	277.87	27.97
qc_C	259.02	253.90	276.16	28.20
qc_C	259.80	258.72	252.61	28.91
qc_C	271.96	261.03	284.84	28.83
**average**	**267.70**	**256.82**	**270.17**	**28.18**
**std dev**	**7.15**	**5.31**	**12.88**	**0.89**
**% CV**	**2.7%**	**2.1%**	**4.8%**	**3.2%**
**% accuracy**	**107.1%**	**102.7%**	**108.1%**	**112.7%**

calibrated with low level standard curve

QC E (100/10)	anabasine pg/mL	anatabine pg/mL	anatalline pg/mL	NNAL pg/mL
qc_E	**109.45**	**110.06**	**102.38**	**9.60**
qc_E	94.13	100.65	107.44	10.44
qc_E	112.59	106.54	110.23	10.99
qc_E	102.81	104.35	117.36	10.69
qc_E	109.72	102.83	100.97	10.18
qc_E	108.22	108.28	106.73	9.79
**average**	**106.15**	**105.45**	**107.52**	**10.28**
**std dev**	**670.9%**	**350.7%**	**590.1%**	**53.2%**
**% CV**	**6.3%**	**3.3%**	**5.5%**	**5.2%**
**% accuracy**	**1.062**	**1.055**	**1.075**	**1.028**

calibrated with low level standard curve

QC D (30/3)	anabasine pg/mL	anatabine pg/mL	anatalline pg/mL	NNAL pg/mL
qc_D	**27.13**	**37.08**	**30.69**	**3.09**
qc_D	27.22	29.15	31.63	3.10
qc_D	27.30	24.94	31.93	3.51
qc_D	31.87	31.43	35.00	3.33
qc_D	24.64	25.08	37.06	3.39
qc_D	26.60	26.08	30.84	3.22
**average**	**27.46**	**28.96**	**32.86**	**3.27**
**std dev**	**238.1%**	**472.2%**	**258.4%**	**16.8%**
**% CV**	**8.7%**	**16.3%**	**7.9%**	**5.1%**
**% accuracy**	**0.915**	**0.965**	**1.095**	**1.091**

calibrated with full standard curve
(9/19/12 pool smoker urine)

pool QC	anabasine pg/mL	anatabine pg/mL	anatalline pg/mL	NNAL pg/mL
SU	**5381.72**	**4795.19**	**2808.87**	**48.53**
SU	5228.56	4716.39	2745.99	46.63
SU	5443.24	4850.11	2746.21	45.95
SU	5133.39	4789.01	2767.67	47.54
SU	5456.89	4859.94	2744.63	47.94
SU	5436.25	4807.32	2774.63	50.88
**average**	**5346.67**	**4802.99**	**2764.67**	**47.91**
**std dev**	**134.29**	**51.45**	**25.11**	**1.72**
**% CV**	**2.5%**	**1.1%**	**0.9%**	**3.6%**
**% accuracy**	**n/a**	**n/a**	**n/a**	**n/a**

4. Table 5 Data
Concentrations of Tobacco Alkaloids and Nicotine Metaboltes in Urine of 20 Smokers

Sample ID #	Anabasine pg/mL (LOQ=100)	Anatabine pg/mL (LOQ=100)	Anatalline pg/mL (LOQ= 50)	Anatalline Metabolite pg/mL (LOQ=500)	Nicotelline pg/mL (LOQ=10)	3'-Hydroxy-cotinine ng/mL (LOQ=10)	Cotinine ng/mL (LOQ=10)
20A-002	14082.5	11941.9	12965.57	4268.56	2249.89	4945.0	1148.9
20A-004	8042.9	6532.8	13214.53	6048.88	3420.27	7724.7	2901.2
20A-005	29568.0	22033.5	25301.22	4016.51	2572.92	9883.2	2045.7
20A-008	6584.3	4106.5	8474.59	1304.50	1038.05	5061.0	1187.3
20A-012	38429.3	33734.5	33515.42	8776.10	6428.78	5454.8	1482.1
20A-015	9869.8	4779.9	10726.85	3909.75	285.73	11514.9	1452.4
20A-019	503.2	508.2	595.41	353.55	95.23	375.9	500.7
20A-024	13125.5	9579.4	6710.97	557.81	525.95	2393.9	1268.2
20A-028	24850.6	22617.2	12976.98	1853.28	747.30	12474.0	2077.8
20A-033	2664.3	1954.4	3068.21	1584.00	432.78	1493.9	1036.4
20A-039	2374.1	2206.8	3563.90	4049.80	1800.13	2714.1	1021.6
20A-042	1218.4	7411.7	8614.52	1412.30	498.07	4274.4	752.8
20A-047	1782.4	1230.1	2282.74	1010.28	370.79	3898.7	687.8
20A-048	3353.5	2856.1	3694.45	1924.89	765.40	7220.9	698.8
20A-050	8424.4	7930.2	4099.72	1167.33	825.07	4628.1	2183.0
20A-055	11236.2	9720.3	11949.48	3187.08	1105.73	7037.5	1481.2
20A-057	23403.3	26043.6	13502.57	3184.02	1852.29	6881.5	2330.3
20A-060	47159.1	27603.2	78126.89	9069.29	4529.06	12546.8	3244.9
20A-062	24275.9	26117.7	26695.37	4656.45	3731.15	10281.7	2338.7
20A-063	10126.1	5700.1	5585.62	739.73	691.13	8351.7	1306.9
**Mean**	**14,054**	**11,730**	**14,283**	**3,154**	**1,698**	**6,458**	**1,557**
**Minimum**	**503**	**508**	**595**	**354**	**95**	**376**	**501**
**Maximum**	**47,159**	**33,735**	**78,127**	**9,069**	**6,429**	**12,547**	**3,245**
**SD**	**13,147**	**10,477**	**17,368**	**2,531**	**1,681**	**3,580**	**763**

Red Font: BLQ and LOQ/Square Root 2 Used

Concentrations of Tobacco Alkaloids and Nicotine Metaboltes in Urine of 19 Non-Smokers

Sample ID #	Anabasine pg/mL (LOQ= 100)	Anatabine pg/mL (LOQ=100)	Anatalline pg/mL (LOQ= 50)	Anatalline Metabolite pg/mL (LOQ=500)	Nicotelline pg/mL (LOQ=10)	3'-Hydroxy-cotinine ng/mL (LOQ=0.1)	Cotinine ng/mL (LOQ=0.05)
NUTS 001	0.0	0.0	0.00	0.00	0.0	0.000	0.084
NUTS 002	276.1	0.0	0.00	0.00	21.2	0.000	0.000
NUTS 003	0.0	0.0	0.00	0.00	0.0	0.215	0.000
NUTS 004	0.0	0.0	0.00	0.00	0.0	0.935	0.122
NUTS 006	0.0	0.0	0.00	0.00	0.0	0.900	0.287
NUTS 007	0.0	0.0	0.00	0.00	0.0	0.200	0.000
NUTS 008	0.0	0.0	0.00	0.00	10.4	0.242	0.000
NUTS 009	113.3	0.0	0.00	0.00	0.0	0.664	0.130
NUTS 010	0.0	0.0	0.00	0.00	0.0	0.000	0.069
NUTS 011	5228.4	0.0	0.00	0.00	58.6	1.423	0.204
NUTS 012	0.0	0.0	0.00	0.00	0.0	0.238	0.090
NUTS 013	0.0	0.0	0.00	0.00	0.0	0.130	0.094
NUTS 015	257.3	0.0	0.00	0.00	12.6	9.834	2.600
NUTS 016	0.0	0.0	0.00	0.00	0.0	0.170	0.056
NUTS 017	0.0	0.0	0.00	0.00	0.0	0.112	0.000
NUTS 018	0.0	0.0	0.00	0.00	0.0	0.555	0.070
NUTS 019	536.4	0.0	0.00	0.00	0.0	0.728	0.000
NUTS 020	165.6	0.0	0.00	0.00	0.0	0.240	0.000
NUTS 021	0.0	0.0	0.00	0.00	44.5	0.109	0.000
**Mean**	**346.2**	**0.0**	**0.0**	**0.0**	**7.8**	**0.88**	**0.20**
**Min**	**0.0**	**0.0**	**0.0**	**0.0**	**0.0**	**0.0**	**0.0**
**Max**	**5228**	**0.0**	**0.0**	**0.0**	**59**	**9.8**	**2.6**
**SD**	**1191**	**0**	**0**	**0**	**17**	**2.20**	**0.59**
**% Detected**	**32%**	**0%**	**0%**	**0%**	**26%**	**84%**	**58%**

If BLQ, 0 was used

5. Table 6 Data
Alkaloids and NNAL, Total and Free, Pooled Smokers' urine

calibrated with full standard curve
pool QC (9/19/12 pool smoker urine) with enzyme:Total

	anatabine pg/mL	anatalline pg/mL	NNAL pg/mL	anabasine pg/mL
SU+	5765.127	2727.221	87.395	6056.110
SU+	5506.395	2898.349	85.368	6064.551
SU+	5663.785	2903.791	88.333	5890.817
SU+	5507.973	2781.293	84.848	5836.071
SU+	5230.250	2994.200	89.819	5734.058
SU+	5501.261	3006.233	88.821	5900.659
**average**	**5529.13**	**2885.18**	**87.43**	**5913.71**
**std dev**	**181.60**	**112.07**	**1.97**	**128.08**

calibrated with full standard curve
pool QC (9/19/12 pool smoker urine) - no enzyme: Free

	anatabine pg/mL	anatalline pg/mL	NNAL pg/mL	anabasine pg/mL
SU-	3961.083	3172.949	44.654	4849.449
SU-	3751.788	2844.285	46.221	4768.659
SU-	4030.759	2871.269	42.415	4812.734
SU-	3670.679	2912.131	43.655	4495.464
SU-	3772.191	3003.207	44.672	4703.795
SU-	3736.755	2892.444	44.677	4605.383
**average**	**3820.54**	**2949.38**	**44.38**	**4705.91**
**std dev**	**141.77**	**122.19**	**1.27**	**134.42**

6. Figure 8 Data
Alkaloid and NNAL Concentrations

Concentrations in pg/mL. Those BLQ are LOQ/Square Root 2

Participant	Arm	Nicotelline	Anabasine	Anatabine	Anatalline	NNAL	Day
1	Abstinence	28.23	70.77	21.21	47.87	12.32	2
1	E-Cigarette	2.83	840.38	198.95	106.87	38.59	2
1	Tobacco	203.12	3,543.21	4,241.27	1,049.66	75.93	2
2	Abstinence	2.83	3,882.57	290.79	112.16	28.41	2
2	E-Cigarette	2.83	14,361.54	5,223.45	114.69	30.13	2
2	Tobacco	436.31	7,789.16	5,509.29	1,498.80	57.55	2
3	Abstinence	2.83	21.21	21.21	93.74	44.40	2
3	E-Cigarette	2.83	388.31	30.35	186.00	76.10	2
3	Tobacco	924.07	6,264.77	9,490.20	2,850.33	194.03	2
4	Abstinence	2.83	290.56	42.09	191.39	19.95	2
4	E-Cigarette	2.83	523.57	187.73	88.70	14.68	2
4	Tobacco	238.27	2,470.25	4,200.07	672.54	66.03	2
5	Abstinence	2.83	492.45	112.62	1,914.82	211.93	2
5	E-Cigarette	2.83	827.55	527.90	1,296.46	150.72	2
5	Tobacco	1,211.90	20,682.61	26,543.21	8,594.13	597.14	2
6	Abstinence	2.83	3,962.94	1,167.24	123.49	5.40	2
6	E-Cigarette	2.83	10,535.31	7,448.14	103.50	4.89	2
6	Tobacco	385.74	7,391.82	7,064.26	1,523.12	30.48	2
7	Abstinence	2.83	352.47	72.62	489.78	58.21	2
7	E-Cigarette	2.83	1,936.44	1,307.48	519.32	74.92	2
7	Tobacco	399.65	11,426.33	12,680.20	2,919.42	223.54	2
8	Abstinence	2.83	21.21	21.21	24.58	3.79	2
8	E-Cigarette	2.83	21.21	21.21	30.89	6.00	2
8	Tobacco	106.19	1,292.82	1,492.83	444.68	32.56	2
9	Abstinence	2.83	27.70	21.21	133.83	30.47	2
9	E-Cigarette	2.83	1,276.95	683.08	355.85	73.62	2
9	Tobacco	720.64	8,443.30	12,517.07	2,828.26	139.92	2

Participant	Arm	Nicotelline	Anabasine	Anatabine	Anatalline	NNAL	Day
10	Abstinence	2.83	80.10	21.21	96.29	41.80	2
10	E-Cigarette	2.83	34.47	21.21	61.54	34.30	2
10	Tobacco	451.63	5,029.00	4,389.39	905.34	69.21	2
11	Abstinence	2.83	79.43	49.33	605.55	90.42	2
11	E-Cigarette	9.88	360.24	143.41	1,723.20	302.77	2
11	Tobacco	1,745.45	17,569.51	22,215.53	5,915.28	583.27	2
12	Abstinence	2.83	21.21	21.21	131.02	27.38	2
12	E-Cigarette	2.83	72.13	32.53	387.80	48.88	2
12	Tobacco	429.71	8,462.96	8,859.54	4,257.81	226.32	2
13	Abstinence	2.83	21.21	21.21	156.09	3.67	2
13	E-Cigarette	2.83	112.09	29.22	320.61	7.74	2
13	Tobacco	145.53	2,636.90	2,258.46	692.71	31.30	2
14	Abstinence	2.83	21.21	21.21	21.21	2.14	2
14	E-Cigarette	2.83	168.60	29.39	48.98	4.30	2
14	Tobacco	111.46	2,054.72	1,723.33	447.30	10.15	2
15	Abstinence	2.83	21.21	21.21	94.42	24.40	2
15	E-Cigarette	2.83	21.21	21.21	163.39	43.10	2
15	Tobacco	215.34	1,927.53	1,812.50	665.33	29.57	2
16	Abstinence	2.83	100.48	21.21	79.18	20.61	2
16	E-Cigarette	2.83	325.53	30.57	101.17	34.36	2
16	Tobacco	119.45	2,578.58	1,414.49	616.17	50.43	2
17	Abstinence	5.42	80.63	45.29	719.42	109.98	2
17	E-Cigarette	4.84	92.68	48.52	1,083.09	126.90	2
17	Tobacco	579.88	13,523.76	11,301.29	4,346.46	263.08	2
18	Abstinence	11.56	177.25	238.95	245.16	55.70	2
18	E-Cigarette	2.83	43.50	22.29	133.15	30.51	2
18	Tobacco	399.74	5,173.20	8,146.52	1,981.67	158.99	2
19	Abstinence	2.83	46.18	32.92	206.86	79.14	2
19	E-Cigarette	2.83	78.78	21.37	183.86	58.33	2
19	Tobacco	629.25	10,108.85	9,414.19	2,858.93	164.18	2

	Nicotelline	Anabasine	Anatabine	Anatalline	NNAL
Mean, Tobacco Arm	497.54	7,282.59	8,172.30	2,372.00	158.09
Mean, e-Cigarette Arm	3.31	1,685.29	843.58	368.90	61.10
Mean, Abstinence Arm	4.76	514.25	119.16	288.78	45.79

LOQ, pg/mL	4	30	30	30	3
LOQ/Square Root 2	2.83	21.21	21.21	21.21	2.12
